# Proceedings of Georgia State Dental Association

**Published:** 1871-05

**Authors:** R. A. McDonald

**Affiliations:** Cor. Secretary.


### ARTICLE III.

### *Proceedings of Georgia State Dental Association.*

By R. A. McDonald, D. D. S., Cor. Secretary.

This Association met on the fifth day of April 1871, in Augusta, the President, Dr F. Y. Clark, of Savannah Ga., in the chair.

The Secretary Dr. S. C. Ford, of Atlanta, Ga., being absent, Mr. Samuel Hape, of Atlanta, Ga., was appointed by the Chair to act as Secretary, *pro tem.*

The following members present answered to their names:

Drs. W. H. Burr, Madison, Ga.; J. P. H. Brown, Augusta, Ga.; E. Parsons, Savannah, Ga.; H. A. Lowrance, Athens, Ga.; A. M. Postley, Savannah, Ga.; F. Y. Clark, Savannah Ga.; E. M. Allen, Marietta, Ga.; S. G. Holland, Agusta, Ga.; Thomas J. Jones, Sparta, Ga.; R. A. McDonald, Griffin, Ga. Drs. S. J. Cobb, of Nashville, Tenn., and G. McDonald, of Aiken, S C., were elected honorary and corresponding members, and Drs. M. D. Jobson, of Perry, Ga.; S. Wright, of Augusta Ga., H. F. Camfield, and A. F. Bignon, Augusta, Ga., regular members. Dr. E. Parsons, Chairman of the Committee on Physiology, Dental Histology, Surgery and Chemistry, read a very interesting and able paper on the subject of Dental Histology and Chemistry, with some very interesting facts in connection with the early history, rise and progress of Dentistry. The report was discussed by Drs. F. Y. Clark, S. J. Cobb, of Nashville, and J.P.H. Brown, of Augusta.

Quite a lengthy discussion was entered into as to the cause of caries and its prevention, participated in by Drs. Clark, Parsons, Cobb and Brown.

Dr. S. McDonald of Aiken, S.C., spoke on the subject of operative dentistry. He had been in the practice of dentistry some forty years. At the time he commenced practicing, great difficulty was experienced in obtaining suitable instruments. A *blacksmith* was his instrument manufacturer. The Doctor stated that some instruments invented by himself at that early stage of his professional career, were still in use, and had been adopted as standard patterns. Gold plate was his favorite *base* for artificial dentures. Has had no experience in the use of heavy foils. Prefers gold foil to any other preparation of that metal for filling teeth. The treatment of alveolar abscesses was also discussed. Dr. McDonald used caustic potash, and treated the teeth by an application to apex by penetrating the seat of the abscess externally.

Dr. Parsons also spoke of the difficulties experienced by the early practitioners of dentistry in getting suitable instruments, &c.; difficult operations were not attempted ; extraction was resorted to where there was any difficulty in filling a tooth. He thinks that amalgam filling is increasing, and that this material is often used where gold would be decidedly better; thinks however, that amalgam can be used to great advantage in some cases. Dr Parsons here presented an instrument for using amalgam to the State society. It is in the form of a *spatula* of silver. The affinity between silver and mercury enables the operator to carry any desired quantity to the cavity, and take up any particle that may accidentally drop in the mouth while packing ; conceived the idea and made the first instrument in August 1870. An interesting specimen of exostosis was here presented by Dr. Parsons. The tooth was extracted some two months since ; a lady had been treated for facial neuralgia by her physician for four months without any beneficial results ; found great difficulty in extracting the tooth with forceps ; finally succeeded with an excavator.

Dr. McDonald called the attention of those present to his inferior incisors which had been separated in 1824 by a dentist in New Orleans.

Dr. E. M. Allen alluded to a subject that had not been treated of, viz : the examination of very small cavities in the fissures of the molar teeth. Had a recent case where a patient had been dismissed by her dentist, who assured her that her teeth needed no attention, and Dr. A. filled nine cavities, upon a thorough examination.—Advises great care in the examination of this class of cavities. He has used sesqui-chloride chromium in cases of very sensitive teeth with beneficial results. Dr. Parsons has used caustic potash with success. Dr. J. P. H. Brown has very little faith in remedies of this kind. He would recommend a very sharp instrument, accompanied with moral influences and suasion. Dr. Allen would like to know if this moral influence can be brought to bear upon all patients. Dr. Brown thinks not; but in many it can.

Dr. F. Y. Clark spoke on the subject of heavy foils; thinks that heavy foil can be used in almost every sort of cavity; can get from one-third to one-fourth more foil in a cavity than with lighter numbers. Also alluded to the use of oxychloride of zinc for capping nerves. It can be used in a very thin state by flouring over the exposed nerves with good results. The prejudice against the use of this material is not as strong as when first introduced; thinks caustic potash should be used with great caution; believes in sharp instruments and moral suasion; when something must be used to destroy sensitiveness, uses oxychloride of zinc.

Dr. Thos. J. Jones, Chairman of the Committee on Mechanical Dentistry, made a verbal report that it is the unanimous opinion of the committee that gold work is in a large majority of cases superior to all other styles of work now in use, and though the first cost is more, that it is the cheapest in the end, and free from many of the objections attending the use of vulcanite work, which is susceptible of many abuses and is too frequently palmed of on persons who have no means of discriminating between the different styles of work.

Specimens of improved impression cups were presented by Dr. G. McDonald, of Aiken, S. C., and, on motion, a vote of thanks was extended and a committee appointed to investigate and report at the next annual meeting.

The following standing Committees were appointed:

*Physiology, Histology and Chemistry*—Dr. A. C. Ford,

Atlanta; Dr. D. S. Wright, Augusta; Dr. A. M. Postley, Savannah.

*Operative Dentistry*—Dr. F. Y. Clarke, Savannah; Dr. A. F. Bignon, Augusta; Dr. M. S. Jobson, Perry, Ga.

*Mechanical Dentistry*—Dr. T. J. Jones, Sparta; Dr. E. S. Billups, Atlanta; Dr. M. H. Thomas, Monroe.

*Dental Education and Literature*—Dr. J. P. H. Brown, Augusta; Dr. E. M. Allen, Marietta; Dr. H. T. Camfield, Augusta.

The Committee on Information for the People then made their report. The article was read, and, on motion, was received, and after some slight alterations, adopted. The Executive Committee was authorized to have 15,000 copies printed, each dentist to pay the expenses of printing for the number of copies purchased.

The following resolutions were adopted successively:

*Resolved*, That a committee of——— be appointed to regulate prices for dental operations. [And after being read and discussed referred to executive committee.]

*Resolved*, That the secretary be directed to furnish the various dental journals with a copy of the proceedings of this meeting.

*Resolved*, That the secretary furnish every dentist in this State with a copy of the By Laws and Constitution of this Society.

*Resolved*, That the Corresponding Secretary be directed to confer with the *American Journal of Dental Science* as to printing the essays and reports of committees of this Society, and report at our next meeting.

On motion the following delegates were appointed to the Southern Dental Convention, meeting in Charleston, S. C:

Drs. H. T. Camfield, W. E. Spears, D. S. Wright, A. F. Bignon, J. P. H. Brown, S. Hape, S. G. Holland, F. Y. Clarke, W. H. Burr, M. S. Jobson, H. A. Lowrance.

It was moved and adopted that a committee be appointed to make arrangements for the next meeting, Drs. Ford, Carpenter and Hape, were appointed as the committee.

On motion, the next place of meeting was decided to be Atlanta, Ga. and the time of our uext annual meeting to be the first Wednesday in April, 1872, 10 o'clock A. M.

The following resolution was unanimously adopted:

*Resolved*, That a vote of thanks be tendered to the Fac ulty of the Augusta Medical College, members of the Press, Hotels, various railroads centering in Augusta, proprietors of the Masonic Hall, and the dental profession of Augusta, for kindness shown and courtesies extended to the Society.

No further business claiming the attention of the Association, the meeting adjourned.

---

### ARTICLE IV.

### *Alabama Dental Association.*

Sec. Ala. Dental Association.

